# Migrant agricultural workers’ deaths in Ontario from January 2020 to June 2021: a qualitative descriptive study

**DOI:** 10.1186/s12939-022-01692-7

**Published:** 2022-07-16

**Authors:** Susana Caxaj, Maxwell Tran, Stephanie Mayell, Michelle Tew, Janet McLaughlin, Shail Rawal, Leah F. Vosko, Donald Cole

**Affiliations:** 1 Western University, London, Canada; 2 University of Toronto, Toronto, Canada; 3 Occupational Health Clinic for Ontario Workers, Hamilton, Canada; 4 Wilfrid Laurier University, Brantford, Canada; 5 York University, Toronto, Canada

**Keywords:** Migrant agricultural workers, Health risks, Mortality, Death, Canada, COVID-19

## Abstract

**Background:**

Nine migrant agricultural workers died in Ontario, Canada, between January 2020 and June 2021.

**Methods:**

To better understand the factors that contributed to the deaths of these migrant agricultural workers, we used a modified qualitative descriptive approach. A research team of clinical and academic experts reviewed coroner files of the nine deceased workers and undertook an accompanying media scan. A minimum of two reviewers read each file using a standardized data extraction tool.

**Results:**

We identified four domains of risk, each of which encompassed various factors that likely exacerbated the risk of poor health outcomes: (1) recruitment and travel risks; (2) missed steps and substandard conditions of healthcare monitoring, quarantine, and isolation; (3) barriers to accessing healthcare; and (4) missing information and broader issues of concern.

**Conclusion:**

Migrant agricultural workers have been disproportionately harmed by the COVID-19 pandemic. Greater attention to the unique needs of this population is required to avoid further preventable deaths.

COVID-19 has given rise to new health risks and challenges throughout the world. Migrant workers employed in high-income countries during the pandemic were often deemed ‘essential workers,’ yet they generally endured high-risk work environments without the health, safety, and economic measures that would protect them should they be exposed to COVID-19 [1]. Mirroring trends in other countries, [2, 3] migrants working in agriculture and food processing in Canada have been disproportionately harmed [4, 5]. Citing long-standing barriers to health, in addition to various social and legal vulnerabilities, several experts and advocates raised the alarm at the start of the pandemic, predicting that this group would be uniquely impacted by COVID-19 [6]. Following the pattern seen among essential workers nationally and internationally, the migrant agricultural workforce experienced higher rates of infection and deaths related to COVID-19 [7]. In the Ontario context, inequities in the burden of COVID-19 among both low socioeconomic and ethno-racially diverse neighbourhoods have been identified [8]. Concurrently, in the United States, COVID-19 illnesses and deaths have been found to be higher in regions with more agricultural workers, including migrants [9]. In several European countries and the United States, the spread of COVID-19 among migrant agricultural workers has been exacerbated by cramped living quarters, with limited access to sanitation, protective equipment, and barriers to accessing testing [10].

From 2020 to 2021, at least nine migrant agricultural workers died in the province of Ontario alone, with additional deaths reported in other regions of Canada. While not all of the nine deaths recorded in Ontario were attributable to a diagnosis of COVID-19, several of these workers died during the initial quarantine period after their arrival into Canada [11]. Accounts of these individuals’ deaths in the Canadian media and by known contacts suggested that inappropriate public health strategies, limited health literacy, isolation, service access and navigation issues, and/or lack of culturally responsive medical care may have contributed to their deaths. In many cases, experts and advocates alike suggested that the deaths of several of these individuals could have been prevented [12]. Yet, many of the details related to these deaths were not made public, limiting what recommendations and strategies could be put forward to aid in the prevention of further deaths.

Our research study sought to better understand the various overlapping factors that contributed to the deaths of identified migrant agricultural workers who died in Ontario between January 2020 and June 2021. The goal of this study was to develop evidence-informed recommendations aimed at preventing future deaths among this population. To this end, we applied a qualitative descriptive approach to our review of all coroner reports related to the deaths of nine migrant agricultural workers in Ontario and supplemented this review with a media scan of the most relevant coverage of each individual’s death. In the discussion to follow, we begin by introducing our theoretical framework and reviewing the literature before reporting the key findings of our study. Considering the continued risks faced by migrant agricultural workers in Canada and elsewhere, we conclude by presenting timely recommendations for policy and service delivery that emerge from our findings. Our recommendations are aligned with the objectives of the UN Global Compact for Safe, Orderly and Regular Migration (GCM), [13] and are guided by the WHO Framework of Priorities and Guiding Principles to Promote the Health of Refugees and Migrants [14]. The GCM outlines specific objectives regarding decent work and health of migrant workers, in addition to key guiding principles, including gender responsiveness, human rights, people-centeredness, and a whole of government and whole of society approach, among others.

Migrant workers encounter occupational hazards, impaired access to healthcare, and human rights’ violations across the globe [15–18]. A recent systematic review of migrant workers’ continuum of care for noncommunicable diseases indicates that language, culture, legal status, and social exclusion are key factors that compound other social determinants of health and ultimately affect migrant workers’ access to and navigation of healthcare services [19]. Our study applies a socioecological model to understanding migrant agricultural workers’ deaths in Ontario, Canada with the intent of yielding recommendations that will help to prevent future deaths among this population, but that will also be of relevance to the broader international community. Such a framework can equip researchers to address health equity issues by guiding an analysis of a wide range of determinants and factors within their environmental contexts [20].

## Theoretical framework and literature review

The socio-ecological model has long been adopted by scholars of agricultural health and medicine, as well as various public health institutions, to capture the complexity of factors and levels of influence that contribute to health environments [21, 22]. This model supposes a convergence of factors that occur at multiple levels to impact a public health issue and is therefore an appropriate model through which to examine the social and ecological complexities related to COVID-19. The spheres of influence in the socio-ecological model include (1) interpersonal conditions, (2) social connections (i.e., relationships), (3) wider community settings (e.g., schools, workplaces, neighborhoods) and (4) broader societal norms, policies and political forces [21, 23]. Applying this lens facilitates interventions at various levels, thereby more effectively addressing health inequities and areas of harm faced by individuals and populations and informing more comprehensive strategies for prevention [21, 24]. In our review of the literature that follows, we will first discuss the labour arrangements of migrant agricultural workers in Canada. We then proceed to examine the different levels of influence of the socioecological model as they can be understood to shape these workers’ health and safety experiences.

### Migrant agricultural workers’ labour arrangements

Typically, migrant agricultural workers are recruited to work in Canada in one of two migrant work programs, the longstanding Seasonal Agricultural Worker Program (SAWP) or the low-wage agricultural stream of the Temporary Foreign Worker Program (TFWP) [25]. Operating continuously since 1966, the SAWP is the largest program employing agricultural workers, and functions based on two bilateral agreements, one between Canada and a group of eleven Caribbean countries, and another between Canada and Mexico. Under the SAWP, workers are allowed to work in Canada for a maximum of 8 months a year [26]; although the program permits circularity, SAWP workers must return to their countries of origin by December 15th of their year of arrival. Prior research suggests that on average SAWP workers return to work in Canada for approximately 10 years, with a good proportion returning to work in Canadian agriculture for several decades, challenging the category of ‘temporary’ in the classic sense of the term [27, 28].

In recent years, a growing number of migrant agricultural workers have been entering Canada under the distinct agricultural and low-wage streams of the TFWP. Under these streams, workers are not recruited or directly represented by authorities in their countries of origin; rather, they are recruited by non-profit and third-party agencies. These individuals may come from any region of the world, but most common among them are workers from Mexico, Guatemala, Jamaica, Thailand, and the Philippines [29]. Under this program, workers may be authorized to work for up to 2 years in Canada for the duration of their contract.

In addition to these formal programs, individuals without authorization to work are also employed in Canadian agriculture, although their numbers are difficult to estimate. There are several reasons that this undocumented workforce exists. Individuals may seek out opportunities to work under tourist visas. Individuals who have faced threats, abuse, or dismissals under a formal migrant program may opt to stay in Canada and “work under the table” as a method to maintain a source of income for their family [30–32]. Some individuals may be recruited to Canada under false promises or misrepresentation of their work conditions in Canada, and only become aware of their lack of formal status shortly after arrival in the country [33, 34]. In such cases, a lack of legal status poses challenges for these workers to access healthcare and other protections [35].

### Interpersonal conditions

While the SAWP and the Low-Wage and Agricultural streams of the TFWP are associated with certain risks and structural vulnerabilities (discussed below), migrant agricultural workers are highly motivated to continue their work in Canada because of limited economic opportunities in their countries of origin. Some individuals come from regions where there is political conflict or instability, while others come from areas where global-scale industries or political policies have endangered their traditional livelihood [36, 37]. Two studies in the Canadian context also indicate that Indigenous-identified persons are over-represented among migrant agricultural workers, making up over 50 to 60% of the SAWP population specifically [38, 39]. Having dependents back home is an informal requirement for entry into such programs in some national contexts; thus, most participants are parents with children, and most women in the program are single mothers [40]. As Canadian laws do not allow reunification of family members among this workforce [41] separation from spouses and children is a necessary reality.

Limited opportunities in home countries as well as the recruitment process for certain programs are such that migrant agricultural workers typically have minimal access to formal education, and some struggle with low levels of literacy [16, 42, 43]. Among the Mexican workforce, who make up the largest subpopulation of migrant agricultural workers in Canada, most are from rural and remote settings with some Indigenous workers not having fluency in the dominant language of Mexico (i.e., Spanish). Most workers from Mexico have limited or no knowledge of the English language. Although Caribbean workers are generally fluent in English, there are significant differences in socio-cultural norms and behaviors that make navigating life in Canada difficult and stressful, and this is especially challenging for those who experience issues related to literacy [44–48].

### Social conditions/relations

In Canada, migrant agricultural workers often live and work in small, rural towns that have limited ethnocultural diversity and very few amenities [49]. Within these regions, infrastructure for public transportation and access to private vehicles is typically inadequate or non-existent [50]. Furthermore, because workers are generally housed in employer-provided accommodations, this group is even further isolated, and may be expected to be “on call” at all times, thus limiting their ability to build connections off of the farm [51]. At the workplace, relationships among peers may be strained because of a sense of competition among co-workers, which is in part fueled by the power imbalance between employers and workers [52]. Since their temporary status and work permit are tied to a single employer, who can fire and deport them without explanation, workers fear deportation and/or not being asked back by their employer. This deportability puts pressure on workers to outperform one another as a means to secure their livelihoods for subsequent seasons as well as to avoid making formal complaints about their conditions of work and health [53]. Some employers explicitly enforce the isolation of their migrant workforce through the imposition of curfews, visitor restrictions, and other measures of control [54]. Adding to this strain, many migrant workers endure threats, abuse or intimidation [44], with recent research finding that one in four workers reports such experiences [38, 42].

Language barriers, lack of familiarity with health and social care services, and limited transportation also make it difficult for workers to access resources and independent means of communication and support services [50, 51]. Access to healthcare and basic requirements, such as groceries and banking needs, are typically contingent on an employer or supervisor [37, 44, 51]. Employers are responsible for arranging trips to town every one or 2 weeks. As a result of this dependence, and because workers are conscious of being evaluated as productive, individuals may feel pressured, or see it in their best interest to minimize symptoms, delay seeking care, or ignore their declining health in order to continue working. Likewise, employers or supervisors may encourage or enforce this behavior if it is viewed as posing a threat or delay in farm operations [48, 55, 56]. Furthermore, when workers seek medical care, they often continue to rely on employers or supervisors for spoken language interpretation, which poses challenges for full disclosure and adequate follow-up in care for both workers and healthcare providers [37, 57]. For instance, employers may discourage workers from reporting workplace injuries, or censor workers’ concerns to a physician [51, 54].

Workers’ experiences of isolation, tension among peers, employer gatekeeping and meddling in terms of help-seeking needs are often exacerbated by poor housing conditions [40, 49, 55]. Typically overcrowded, lacking in privacy and ventilation, and with minimal regulation and oversight by authorities, employer-provided congregate housing has been identified as a key source of SARS-CoV-2 transmission among migrant agricultural workers, and a general risk to workers’ health and safety [58, 59]. Poor housing conditions may also send clear messages of disrespect and lack of dignity for this population that can exacerbate a sense of hopelessness and futility when it comes to protecting one’s rights in Canada [50, 60].

### Wider community settings

As previously mentioned, migrant agricultural workers are generally housed on or near their employer’s property, and thus typically geographically and socially isolated from the wider community. Local communities may be hostile to this workforce, because of xenophobic rhetoric that portrays migrant workers as “job thieves,” or as vectors of disease, especially in light of new fears and uncertainties as a result of COVID-19 [61]. For instance, in the region where the first COVID outbreak among migrant agricultural workers took place, British Columbia, workers reported being racially profiled and policed in grocery stores as a result of their racialized status and questioned about whether or not they worked on the particular farm where an outbreak took place [55]. Unsurprisingly, migrant agricultural workers report a limited sense of belonging while in Canada [42, 50, 62]. Caribbean workers in particular have been victims of explicit racial profiling and anti-Black racism [43, 45].

Furthermore, prior research indicates that migrant agricultural workers expect to get inferior healthcare in comparison to their Canadian and permanent resident counterparts [42]. Migrant workers have also reported that clinicians do not understand how their health status can affect their [43] employment in Canada [37, 42, 51]. This lack of understanding is notable given that incidences of medical deportation among this population are well-documented [63]. Many migrant workers report that their privacy is not maintained in healthcare settings. Among non-English speaking workers, many report that they are not provided with an independent interpreter while receiving care, and instead have to rely on their employer for spoken language interpretation. Our prior research with clinicians further indicates that healthcare workers often do not understand what benefits and services this population is entitled to, and thus, adequate processes are not in place to report workplace injuries and other claims that all workers in Canada are entitled to [37]. Since most migrant agricultural workers are not familiar with formal legal and healthcare system processes, most are unable to advocate for themselves, whether in regards to occupational health and safety, healthcare services, or human rights abuses [38, 42, 64].

### Broader social and political context

Even when workers are able and willing to advocate for their rights, prior research indicates that this population has limited faith in the responsiveness of both Canadian and sending country authorities to respond to concerns or threats they identify while working in Canada [42]. Sending country officials tasked with representing their migrant workforces are generally poorly staffed, and their loyalties may be divided as a result of their responsibility to maintain high enrollment of program participants from their countries of origin [36, 65]. This may result in country-of-origin officials encouraging participants to not complain, or smoothing conflict between employers and workers [51]. At a more collective level, there have also been incidents of migrant agricultural workers being blacklisted by their nation-state officials for engaging in labor organizing activities [66].

The regime tasked with overseeing the working and living conditions of migrant agricultural workers in Canada is also a source of vulnerability. A recent report by the Auditor General of Canada notes that inspections carried out by federal officers in 2020 found problems with 73% of the quarantine inspections they examined, and that this number rose to 88% in 2021 [67]. Consistent with research findings on the federal inspection regime predating the pandemic, [68] the Auditor General found that employers were assessed as being in compliance in the absence of any evidence at all. This report follows many other lack-of-oversight issues that have been documented by researchers, including: (1) an inadequate amount of unannounced inspections, which enables employers to misrepresent living and working conditions; (2) an over-reliance on complaints by migrant agricultural workers, a group that is hesitant to report, and made vulnerable should they step forward; and (3) a lack of anti-reprisal mechanisms to protect workers who identify abuses or violations [69–71]. The consequence of all of these regulatory challenges is that many workers may think that it is futile or dangerous to report concerns or lack the necessary support to make a complaint.

The precarious conditions under which workers labor in agriculture, coupled with the conditionalities attached to Canada’s migrant worker programs in this sector, pose significant obstacles for migrant workers’ health status and protection. As noted, individuals in these programs are tied to a single employer who can fire and deport them at will, and yet they rely on employer nominations in order to return to the same workplace, and even to return to the program the following year [72, 73]. This creates a “perverse incentive” to work beyond one’s physical limits in an effort to guarantee re-employment [74]. Furthermore, because migrant employees in agriculture cannot simply opt for a different employer should they find their living or working conditions inadequate, they may be less likely to refuse unsafe work and housing conditions [51]. Workers’ temporary residency status alone can limit their ability to seek entitlements or make claims to which they are entitled, both for logistical and legal reasons. For example, making claims for workplace injuries, necessary medical follow-up, or accessing justice when facing abuses, may be interrupted by a worker’s repatriation to their country of origin, or permits that ‘expire,’ posing legal and financial consequences should workers choose to persevere [75]. For undocumented workers or those unauthorized to work, their knowledge and access to healthcare and labour rights is even more tenuous, [76] and may be made more complicated by experiences of labor trafficking or recruitment agency exploitation [77–79]. In sum, this population’s partial or limited status in Canada as temporary migrant workers subjects them to additional health risks and limits their ability to access rights and protections.

## Methods

We employed a modified qualitative descriptive methodology. This approach is well-suited to the development of a straightforward description of a topic. In turn, an accessible rendition of findings can be taken up easily to inform future interventions [80, 81]. Such a methodology is intended to study phenomena as they naturally unfold, rather than in a controlled environment, and to answer “what, where, and who” questions. Furthermore, while this approach can be theory-driven, it affords researchers the flexibility to adapt different processes and interpretations to ensure the comprehensiveness of the findings. Our key research questions were:

1. What was the medical trajectory of the migrant agricultural workers who died in Ontario, Canada in 2020/2021?
2. What shared conditions/ issues were experienced/lived by these individuals? How can these experiences help us understand the death of these individuals?
3. How can these findings support points of intervention and prevention that may decrease the risk of death and serious illness or impairment among migrant agricultural workers?

We were interested in examining the trajectories of the migrant agricultural workers who died in Ontario during the COVID-19 pandemic from 2020 to 2021. Therefore, we relied on the Office of the Chief Coroner’s files related to nine individuals who died during this period. To supplement this dataset, we carried out a media scan. This scan was carried out by reviewing the first five pages of hits on a popular search engine of each worker’s death. Key search words included migrant, farmworker, death, country of origin and related terms and limiters of Ontario, Canada. This provided us with additional information about each worker’s death that had been publicly known and covered by mainstream media, albeit to varying degrees.

Together with a content analysis approach, our research process was intended to keep our findings descriptive and informative rather than interpretive, especially regarding questions 1 and 2. Towards this aim, we developed a standard extraction tool. Components of the extraction tool included structured questions related to: (a) the type of files made available; (b) demographic information, including previous health history; (c) cause, manner and specifics related to the individual’s death; (d) length of illness, type/degree of healthcare received, presenting symptoms, and any other presenting health issues; (e) travel details, including travel route; (f) occupation/workplace details, including number of co-workers and location; (g) several data points related to COVID-19 vaccination history, including adverse events; (h) details of initial and subsequent quarantine period, including location, living quarter characteristics; (i) types of supports and resources provided, including translation, telemedicine and employer’s role; and (j) proximity to healthcare centres, degree of medical check-ins. Furthermore, in keeping with the socio-ecological model and prior research on migrant agricultural workers, several open-text boxes were included to be able to fully document anything of relevance to our research topic at the interpersonal, social/relational, community, and structural/broader contextual levels. Each file was reviewed by a minimum of two readers, with the most complex files reviewed by three readers. These reviewers, each with clinical backgrounds, collectively brought expertise in occupational health, community mental health, public health, and infectious diseases. Separate readings of each file were merged to ensure maximum comprehensiveness of the extraction. The few discrepancies that were noted were flagged for further review and addressed through further discussion by all reviewers.

Following the initial coding process, our research findings were presented to a panel of experts, including staff members in the coroner’s office. At this point, initial recommendations and areas of action were also provided in draft form, to support further dialogue, and to refine our findings to answer the third research question. Our team returned to the initial data and codes as necessary to address questions or feedback provided by our peers. Direct quotes from the coroner files are not included given the technical nature of the text available for review was not conducive to a qualitative presentation of findings, and because the specificity of written information could easily have revealed identifying information about an individual.

This project was approved by the ethics board at the first author’s institution, Project ID: 119421 and by the Chief Coroner’s Office under a research agreement between the principal investigator and the office. All extracted data were anonymized upon review, with original files remaining under the possession of the coroner’s office. A legend was kept in a separate location from extracted data with respective codes and identifying information and was only made available to the research team. All data were encrypted and stored on a secure local server hosted by the first author’s institution.

## Results

Between 2020 and 2021, there were at least nine deaths among migrant agricultural workers in Ontario. A review of the coroner files of the nine individuals confirmed to be migrant agricultural workers revealed that all the deceased were male (reflecting a male-dominated population). They ranged in age from 22 to 57 years old, with three workers in their 20s, and most individuals presenting with no previous health issues of concern. The workers had arrived in Canada from across Central America and the Caribbean, with the majority arriving from Mexico [Table 1].

**Table 1:** Characteristics of migrant agricultural worker deaths

Characteristic	Proportion
Sex	
Male	9/9
Country of origin	
Mexico	5/9
Caribbean	3/9
Central America	1/9
Cause of death	
Related to SARS-CoV-2 (i.e., primary or secondary contributing factor)	6/9
SARS-CoV-2 pneumonia	3/6
Other	3/6
Myocardial infarction	1/9
Motor vehicle accident	1/9
Unknown	1/9
Quarantine status at time of death	
Initial quarantine period	4/9
Timing of death in relation to positive test for SARS-CoV-2	
< 1 week	2/6
1–2 weeks	1/6
2–3 weeks	1/6
> 3 weeks	2/6
Timing of death in relation to onset of SARS-CoV-2 symptoms	
< 1 day	3/6
> 1 day	2/6
Unknown	1/6

Findings from the coroner file review and subsequent media scan yielded four broad domains of risk: (1) recruitment and travel as sites of vulnerability; (2) missed steps and substandard conditions of healthcare monitoring, quarantine, and isolation; (3) barriers to accessing healthcare; and (4) missing information and broader issues of concern [Table 2]. Each of these domains encompassed various factors that either likely exacerbated the risk of poor health outcomes, and/or directly contributed to the medical trajectory of the deceased workers. While many of these challenges and barriers faced by migrant agricultural workers have been documented across the international literature, [19] our study is among the first to identify these factors as key contributors to the deaths of a cohort of workers.

1. ***Recruitment and travel as sites of vulnerability***

**Table 2:** Summary of key findings and recommendations from coroner file review and media scan

Domain	Themes	Examples	Recommendations
Recruitment and Travel Risks	Exploitative recruitment	• One worker arrived in Canada after third-party recruiters had misrepresented his employment, leading to debt and illegal deductions and poor/crowded housing conditions that could not be reported	• Legislators should set out the duties of agencies/recruiters under the Occupational Health and Safety Act.
	Travel as a risk factor for transmission	• Several workers became ill with COVID after initially testing negative on arrival, suggesting that they became infected during travel to Canada, transportation to farm/quarantine accommodations, or during the initial quarantine • In several cases, workers entered Canada twice in the same year to work at different farms	• Implement measures to minimize exposure to SARS-CoV-2 during travel, taking into account the significance of airborne transmission. These measures should include the provision of N95 or KN95 masks, or equivalent, with instructions on their use. • Provide accessible, independent mobile testing for migrant agricultural workers at regular intervals during quarantine.
Missed Steps and Substandard Conditions of Healthcare Monitoring, Quarantine, and Isolation	Precarious housing and living conditions	• Workers often shared accommodations with several other workers, especially during the initial quarantine period • A worker who died of motor vehicle blunt trauma while working/travelling at night on a dirt road	• A national housing standard should be established and enforced, aligned with recognized international standards. • Clear and consistent guidelines should be put in place for the quarantine period that allow for physical distancing, including robust standards that account for the size of living quarters and common areas. • High standards for ventilation (i.e., open windows, HEPA filters, maintenance of HVAC systems, etc.) should be developed, communicated, and enforced. • Regular oversight and adequate investment should be provided for public health units to perform timely, in-person, unannounced housing inspections. • There should be investment in regional transportation plans that account for migrant agricultural workers, and seek to address isolation faced by this group, and also provide road safety infrastructure to protect this workforce from nearby traffic.
	Inadequacy of medical check-ins	• Many workers had a very rapid deterioration after testing positive for SaRS-COV-2 or first demonstrating symptoms • Several workers died during quarantine or isolation • Check-ins were sometimes delegated to employers without medical expertise • One worker was found dead during quarantine with several over-the-counter medications, likely provided to them by the employer	• Workers in quarantine should receive standardized health assessments. These assessments should examine both objective (e.g., temperature reading, pulse oximeter) and subjective (e.g., sore throat, chest pain, shortness of breath) indicators of health status, involve direct communication with the migrant agricultural worker (rather than an employer), and be performed at regular intervals by healthcare professionals with professional interpreters and/or trusted support persons/specialized staff with a track record of working with migrant agricultural workers.
Barriers to accessing healthcare	Challenges seeking emergency care	• When one worker fell ill, workers in another bunkhouse reached out to academics that they had met during a previous growing season (thousands of miles away) to help them call an ambulance • An ambulance was dispatched to the wrong location for an ill worker, delaying medical attention by over 40 minutes	• Workers’ addresses should be clearly posted in their place of residence, along with instructions in their preferred language(s) of how to call emergency medical services.
	Lack of language concordant care	• A worker initially declined intubation (a potentially life-saving intervention) due to a misunderstanding that he would have to pay for it	• Workers should be reassured, in culturally appropriate ways (i.e. in languages and terms that they understand), that emergency care, especially in the COVID-19 context, is not associated with a fee or loss of employment. Any existing fees for emergency treatment that are currently not free of cost for this workforce should be systematically waived.
Missing info raising questions	Referenced medical documentation	• Many coroner reports did not contain copies of referenced medical documentation or contact tracing information to better ascertain source of viral transmission.	• Consideration of this population’s social/geographic isolation, language barriers, international travel, and workplace and living conditions should be part of death reporting. Attending clinicians and coroners should work together to identify and document these factors to ensure relevant issues are identified in the death investigation. A social determinants of health framework may be useful to adopt in the reporting of each death, given the various structural vulnerabilities faced by this group.
	Workers’ compensation	• It was also unclear whether families of deceased workers had a clear path to accessing eligible compensation through the provincial workers’ compensation board.	• Workers, and, when deceased, their next-of-kin, must be made aware of WSIB eligibility, and be given the opportunity to connect with legal advocates who can assist with WSIB applications.

Within this category, we identified several conditions that heightened this group’s likelihood of exposure to SARS-CoV-2. Travel and migratory status, in particular, made it more difficult for individuals to secure safe and healthy working and living environments. Key themes within this domain included (a) exploitative recruitment, and (b) travel as a risk factor for transmission of SARS-CoV-2.

- *1a. Exploitative Recruitment*

One worker was not formally employed under either migrant work programs; rather, this worker arrived in Canada after third-party recruiters had misrepresented his employment. A falsely advertised package that promised English lessons, transportation, and free access to tourist destinations, lured the individual to Canada under a tourist visa, without authorization to work. Once in Canada, the worker needed money to finance his stay in Canada. As a result of poor pay, illegal deductions, and unreliable hours of work offered by private contractors, the worker incurred debt. Furthermore, co-workers facing the same living and working conditions as this individual reported inadequate and crowded housing conditions that they were reticent to report because of their financial vulnerability and lack of authorization to work.

- *1b. Travel as a Risk Factor for Transmission of SARS-CoV-2*

Travel to Canada involves multiple possible routes of exposure, including shared vehicle travel in the country of origin, time at an airport and an international flight, and further shared vehicle transportation to the quarantine or worksite in Canada. Several workers tested positive for SARS-CoV-2 soon after arriving in Canada despite initially testing negative on arrival, suggesting that they may have become infected during travel to Canada, transportation to farm/quarantine accommodations, or during the initial quarantine. For instance, one worker tested negative for SARS-CoV-2 on arrival, then tested positive 10 days later, after sharing a room with three other workers during quarantine. In several cases, workers entered Canada twice in the same year to work at different farms, increasing their risk of exposure.

2. ***Missed steps and substandard conditions of healthcare monitoring, quarantine and isolation***

In investigating the medical trajectory of the deceased individuals, inconsistencies in terms of monitoring, quarantine conditions, and isolation quarters were frequent, suggesting lapses in oversight and standardization. Concerningly, several of the individuals in this dataset did not make it to the hospital for treatment, with their health status declining rapidly resulting in their death. The untimely deaths of these individuals often occurred within a broader context of isolating and substandard living conditions, which sometimes played a determining role in the individual’s medical trajectory. Key concerns under this domain were (a) precarious housing and living conditions and (b) inadequacy of medical check-ins.

- *2a. Precarious Housing and Living Conditions*

Common among the files reviewed was the issue of substandard, inconsistent, and poor housing conditions. Workers often shared accommodations with several other individuals, especially during the initial quarantine period. One worker shared a quarantine accommodation with five other workers. There were reports of crowded, poorly ventilated, and unsanitary living conditions, posing challenges to proper social distancing. Some workers were not provided with sufficient access to personal protective equipment, even after a state of emergency had been declared in Ontario. Notably, remote neighborhoods with dark roads may have also created dangerous working conditions for workers. One worker died of motor vehicle blunt trauma while working near a poorly lit road in a rural area. Previous research has documented other migrant workers dying in a similar manner [47].

- *2b. Inadequacy of Medical Check-ins*

Several workers had a very rapid deterioration after testing positive for SaRS-COV-2 or first demonstrating symptoms. Several workers died during quarantine or isolation, and check-ins were sometimes delegated to employers who lacked medical expertise. A previously healthy worker developed a headache and fever during isolation after testing positive. The next day, the worker called emergency services for shortness of breath. Paramedics found him unresponsive. This was despite reported daily check-ins by a farm employee through the motel room door. Another worker was found dead during quarantine with several over-the-counter medications, such as acetaminophen, ibuprofen, and cough syrup, in his possession, which the employer had likely provided to help manage his symptoms.

3. ***Barriers to Accessing Healthcare***

Under this domain, we identified several barriers for workers not only accessing timely medical care, but also being provided with adequate orientation, both in terms of language and health literacy, to seek help or consent to treatment in a timely manner. Key concerns in this regard included (a) challenges accessing emergency services and (b) a lack of language-concordant care once in hospital.

- *3a. Challenges seeking Emergency Medical Care*

Workers often faced challenges navigating the provincial healthcare system, particularly when it came to accessing emergency services. For example, when one worker fell ill, workers in another bunkhouse reached out to researchers who they had met during a previous growing season (thousands of miles away in another province) to help them call an ambulance. For another ill worker, an ambulance was dispatched to the wrong location, delaying medical treatment by over 40 minutes.

- *3b. Lack of Language-concordant Care*

Access to language-concordant care from healthcare providers proved to be an issue as well. One worker developed a severe case of COVID-19 pneumonia and had poor oxygen saturation on maximal non-invasive ventilation. He initially declined intubation (a potentially life-saving intervention that could provide mechanical ventilation) due to a misunderstanding that he would have to pay for it.

4. ***Missing Information Raising Questions***

Our research identified several concerns involving death reporting and workers’ compensation, with the major theme being missing information within the coroner files of the deceased workers. Key concerns in this regard included (a) referenced medical documentation and (b) workers’ compensation.

- *4a. Referenced Medical Documentation*

Many coroner reports did not contain copies of referenced medical documentation or contact tracing information to better ascertain the source of viral transmission. Several workers had extensive healthcare system interventions, including multiple assessments and investigations, such as bloodwork and imaging, but the source materials were not available to the Deputy Coroner and hence to us as a research team. Likewise, no files made any mention of resources or referrals that had been made to provide individuals with effective lines of communication, or to support their ability to navigate and access the healthcare system.

- *4b. Workers’ Compensation*

In Ontario, migrant agricultural workers are eligible for workers’ compensation benefits if they become ill or injured at the workplace, as well as in employer-provided living quarters, which includes quarantine accommodations, and in transportation between the two locations. However, it appeared that protocols were not in place to either investigate these deaths as work-related incidents, nor to ensure that families of deceased workers had a clear path to accessing eligible compensation through the provincial workers’ compensation board. There was only one instance where the coroner file included correspondence between a worker’s family and an embassy liaison who helped the family access post-mortem reports to file a compensation claim. In some files, correspondence between the Ministry of Labour was available in the files, and a staff member had indicated that investigating the death was considered outside of the mandate of the Ministry because the individual “had not reported to the worksite.” In these cases, workers were in their mandated quarantine following their arrival to Canada.

## Discussion

Overall, our findings suggest that the deaths of the nine individuals under consideration were potentially preventable. Each domain of risk raises important areas for action in terms of policy changes, practice improvements, and further research. Considered through the lens of the socioecological model, these findings also provide guidance on points of intervention at the interpersonal and social/relational level (e.g., need for language concordant care, two-way communication with healthcare professionals) and community and broader structural levels (e.g., addressing xenophobia, deportability, temporary status, and inadequate public health responses). We discuss these issues further below.

### Recruitment and travel as sites of vulnerability

Given that travel is a significant risk factor for transmission, [82] measures to minimize exposure to SARS-CoV-2 that consider the significance of airborne transmission must be implemented [82, 83]. These measures should include the provision of N95 or KN95 masks, or equivalent, with instructions on their use. Migrant workers should also be provided with accessible, independent mobile testing carried out by healthcare professionals at regular intervals, especially during periods of isolation and quarantine. They should have access to testing strategies that match their risk and symptoms, including rapid antigen tests and PCR tests, as appropriate. Efforts to provide migrant workers with access to COVID-19 vaccines should include advance notice, accessible information, the opportunity to consult with a health professional, and language assistance. Going forward, it is necessary for public health officials to plan for preventative measures both to ensure early detection of SARS-CoV-2, and to begin to develop strong lines of communication with this population, who understandably often lack familiarity and trust in the healthcare system [37].

As indicated in the files, and in prior research, climates of coercion and structural vulnerability make it difficult for workers to know about, and even more so, to assert their rights especially when lacking authorization to work in Canada [34, 54, 59, 74, 84]. Yet recruiters’ obligations are poorly defined, and limited mechanisms are in place to oversee their practices. To begin to address this issue, duties of agencies/recruiters must be clearly defined. This change may help make agencies/recruiters and other parties to triangular relationships liable for their conduct. Likewise, even for workers who are recruited through and tied to formal migrant worker programs, their justifiable reluctance and additional barriers to reporting abuses have been well-documented [51]. Therefore, further investment into proactive inspections, anti-reprisal mechanisms, and necessary access to housing and alternative work arrangements, must be put into place [53]. Current options, such as applying for an Open Work Permit for Vulnerable Workers when an individual is facing abuse, are unreliable and involve lengthy mechanisms that do not address the various barriers workers face when pursuing justice [84].

The fear or threat of return through deportability is well established as a source of risk for migrant agricultural workers, as it can force individuals to conform to workplace and living conditions that otherwise might be seen as unacceptable [85]. These findings illustrate that the COVID-19 pandemic introduced risk through *entry* as well as through the threat of return, adding further conditions of risk, and even acceptance of intolerable risk for this population. Revisiting concepts such as deportability, [85, 86] relentless border walls and circular migration, [87] may be important to further explore the relationship between migrant agricultural workers’ health, safety, and the pernicious disciplinary power of mobility, especially within the context of COVID-19.

### Missed steps and substandard conditions of surveillance, quarantine, isolation

Precarious housing and living conditions faced by migrant agricultural workers have been well documented in the literature [49, 88–90]. Our findings indicated that these contexts likely contributed to the deaths of several individuals by heightening the risk of exposure to SARS-CoV-2, limiting access to timely medical care, and generally presenting as unsafe environments. To address substandard living conditions, governments must establish and enforce a national housing standard for MAWs aligned with recognized international standards such as the Guidelines for the Implementation for the Right to Adequate Housing (2019) provided by the UN Special Rapporteur on adequate housing [91]. Specific to the quarantine period, robust standards that allow for physical distancing and account for the size of living quarters and common areas are required. High standards for ventilation (i.e., open windows, HEPA filters, maintenance of HVAC systems, etc.) should be developed, communicated, and enforced. Moreover, regular oversight and adequate investment should be provided for public health units to perform timely, in-person, unannounced housing inspections. Investment in regional programming that accounts for migrant agricultural workers’ transportation needs, road safety infrastructure to protect this workforce from nearby traffic, and initiatives to address their broader isolation from the wider society are also needed.

Divergent and inconsistent experiences with healthcare monitoring, quarantine and isolation among migrant workers speak to the need for a standardized and systematic approach when clinicians are monitoring or providing care for this population within the COVID-19 context. Adopting this approach requires adequate investment into the public health system, and ideally, dedicated teams to build rapport and ongoing relationships with migrant agricultural workers, especially in regions where larger numbers of them are concentrated. Health assessments of migrant workers in quarantine should examine both objective (e.g., temperature reading, pulse oximeter) and subjective (e.g., sore throat, chest pain, shortness of breath) indicators of health status, involve direct communication with the worker (rather than an employer), and be performed at regular intervals by healthcare professionals with professional interpreters and/or trusted support persons and community-based organizations with a track record of working with this population. Prior research has highlighted the importance of dedicated supports to help migrant workers navigate health, social and legal services. Examples of effective approaches include Migrant Health Centres in the USA [17]. These actors can help orient clinicians and migrant workers alike to common challenges faced by this population, help address common structural and individual-level barriers, and empower migrant workers to advocate for themselves, rather than depend on their employers for assistance [92].

The improvement of quarantine and healthcare surveillance measures requires careful consideration of the unique social position of migrant workers in the countries and regions where they work. For instance, during the COVID-19 pandemic, racist and xenophobic sentiments to migrant workers increased throughout the world, with both policy and community members sometimes suggesting that this workforce was the source of risk, rather than they themselves being at risk [93]. In the Canadian context, employers sometimes restricted migrant workers to their living quarters well past their quarantine period, violating their rights to movement or to fresh air under the guise of health and safety [94]. The inadequate public health measures and actions taken for protecting this group can be considered forms of institutional racism with profound bioethical implications [95]. Further research could consider the role of institutional racism in shaping responses to the pandemic, especially as it has affected historically marginalized populations such as migrant workers who face deadly consequences as a result.

### Barriers to accessing healthcare

Clearly, many of the workers who died in 2020 and 2021 faced difficulties accessing timely medical care. This is consistent with accounts from research in the USA [17]. Of the individuals whose deaths we studied, those who did receive care in hospital sometimes faced significant barriers to navigating and advocating for adequate care that suggest several policy changes, especially in the Ontario context. To help orient workers to the process required to call for emergency medical help, addresses must be clearly posted in workers’ place of residence, along with instructions in their preferred language(s) of how to call emergency medical services. To mitigate the risk of language-discordant care, workers should be reassured, in culturally appropriate ways (i.e., in languages and terms that they understand), that medical care, especially in the COVID-19 context, is not associated with a fee or loss of employment. Any existing fees for emergency treatment that are currently not free of cost for this workforce (e.g., ambulance services fees) should be systematically waived. Due to common barriers faced in accessing health care by migrant farmworkers in different regional and national settings, these recommendations are widely applicable.

These incremental steps should be complemented with a greater transformation of the healthcare system, to demonstrate better understanding of the unique barriers faced by this population, and a stronger commitment to anticipate barriers, and deliver culturally appropriate and language-concordant care. As previously mentioned, workers lack faith in the healthcare system and trust in clinicians to help them navigate the complexities posed by their work status [38, 42, 51]. By training front-line care providers to better understand the circumstances and barriers that are faced by this group, and stronger partnerships with support organizations with a track-record of working with this population, improvements in care and overall social support and legal protection for this population can be delivered.

Prior research applying the socioecological lens with diaspora populations indicate that an over-emphasis on individual and interpersonal factors, without consideration of broader intersecting community and organizational factors, can limit necessary transformative action towards health equity [96, 97]. Specifically related to access to healthcare, a socioecological lens can guide health and social care service providers and leaders to improve organizational policies and funding allocations that currently present as obstacles for migrant agricultural workers’ care. Migrant workers’ legal standing as temporary workers, with limited protections and entitlements under the law, also merit scrutiny as factors that contribute to this group’s marginalization, and subsequent risk of death, due to a lack of timely and independent access to services [98–100]. Similar conclusions have been drawn in other regions of the world as a result of increased deaths among migrant worker populations [101].

### Missing information raising questions

The coroner’s files lacked medical documentation as well as information on contact tracing. This was expected because of privacy laws that restrict the coroner’s office from storing medical information. Nonetheless, these gaps in information raised many questions in terms of workers’ medical trajectories and exposures to SARS-CoV-2. Furthermore, limited information was provided about the language barriers, international travel, workplace and living conditions of, and resources/supports available to, the deceased workers. Only a few files made mention of an investigation by the Provincial Labour Ministry, and in some cases, the deaths appeared to have been judged ineligible for investigation by this agency because the worker had died during their initial quarantine.

Future research and policy development should engage multiple agencies to get a clearer picture of the trajectory of migrant agricultural workers’ deaths with the support of investigating coroners. Adopting a social determinants of health framework [102] when reporting deaths among this population may also yield more insight into preventative factors, given the various structural vulnerabilities they face. The Ministry of Labour, and other relevant agencies, regardless of jurisdictional challenges, must develop clear and inclusive parameters to investigate the deaths of migrant workers, whose sole reason for entry into a country is to work. Further, in the case of the files reviewed, all either died or first experienced a decline in their medical status in employer-provided accommodation or on their property. This finding may have significant applicability globally since the vulnerabilities posed for workers living in employer-provided housing have been documented in the international literature [103–105]. This shared feature across many migrant worker groups may serve as an important point of analysis to further understand the unique health and mortality risks faced by this population.

A lack of documentation of the steps taken to communicate with the deceased workers’ next-of-kin regarding workplace compensation raises concerns. Prior research throughout the world indicates that migrant agricultural workers are often unaware of their entitlements for workplace compensation, lack knowledge about how to initiate a claim, or may be discouraged from doing so by authorities or employers [42, 44]. Likewise, it is plausible that these same barriers were at play for families of the deceased. Reliable channels for culturally and linguistically appropriate communication between this workforce and provincial workers’ compensation boards must be systematically initiated, including direct communication with next-of-kin when a migrant worker dies. A specific agency/organization should be tasked to liaise with these agencies, next-of-kin, and if the family wishes, legal advocates to confirm whether coverage is applicable. Such organizations can also support next-of-kin through application processes to workplace compensation boards. The coroner’s investigation can serve as a final appraisal that such mechanisms have been put in place. At a broader level, provincial ministries of labour, agriculture and health must all work closely with national governments to ensure that this precarious workforce is afforded the protections and entitlements for which they are eligible. Ideally, these protections and supports can serve to deter future deaths, and should future deaths occur, ensure a more respectful and dignified response for the families that these individuals leave behind.

## Limitations

Despite close collaboration between the research team and the Office of the Chief Coroner for Ontario, a key limitation of this work is that some migrant agricultural workers may not have been properly identified as such or may have been undocumented; therefore, it is likely that deaths may have been missed (although we were able to include at least one undocumented worker in the study). Another limitation is that our research team did not have access to contact tracing information or primary medical documentation, leading to questions around workers’ potential sources of exposure to SARS-CoV-2 and medical trajectories. In some cases where workers were in quarantine or isolation, it was also unclear who conducted medical check-ins, how often, and what these assessments entailed. To complement our coroner file review, we conducted a media scan that provided additional, publicly available information about each worker’s death, which helped to address some of the above information gaps. Future research into the deaths of migrant agricultural workers should examine primary healthcare documentation, workplace bereavement, workplace safety, and other co-investigative agency documents that may further capture mechanisms and factors of relevance to the deaths of both documented and undocumented workers. In addition, this study was descriptive rather than explanatory in nature. A study of international scope, which can draw from coroners’ investigations or their equivalents among this population in different parts of the world, could likely explore potential explanatory models more fully as has been done with the broader migrant populations in different regions [106]. Yet, our systematic approach provides an important foundation for future action to prevent deaths among a marginalized and underserved population, for whom the pandemic only exacerbated preexisting vulnerabilities.

## Conclusion

This study has sought to describe the experiences, and identify key factors that contributed to the medical trajectory of nine migrant agricultural workers who died in 2020–2021 in Ontario. Using a qualitative descriptive study design, informed by a socioecological framework, we identified four domains of risk: (1) recruitment and travel risks; (2) missed steps and substandard conditions of healthcare monitoring, quarantine, and isolation; (3) barriers to accessing healthcare; and (4) missing information and broader issues of concern. Each of these four domains speaks to the need for strong interventions to protect the lives of this population. Recommendations include independent, mobile SARS-CoV-2 testing; the establishment of national housing standards; proactive enforcement to identify unsafe or substandard living conditions; anti-reprisal mechanisms to empower workers to report concerns; improved orientation for workers on how to access emergency services; language concordant healthcare services; and improved death investigations, with mechanisms for families of deceased workers to access eligible compensation.

Broadly speaking, these recommendations highlight the importance of relationships with public health, the healthcare system, support organizations for migrant agricultural workers, and other key stakeholders and emphasize the need for proactive approaches in partnership with this vulnerable group. Applying a socio-ecological lens, we were able to consider equally (1) individual/interpersonal factors such as the fact that these individuals typically had no prior medical history and were relatively young; (2) social/relational factors, such as a lack of language-concordant care; (3) community/organizational factors, such as a normalization of delegating quarantine check-ins to employers; and (4) broader/structural level factors, such as employer-provided housing, familial separation and temporary and/or marginal status. Ultimately, greater action is needed to reduce the barriers for, and build trust with, this workforce, who have already experienced too many preventable deaths.

Although the specific findings and recommendations were made with the Ontario, Canada context in mind, they can be extrapolated to other jurisdictions. Structural vulnerabilities, including precarious migration and work status, familial separation, poor living and working conditions, and systemic barriers to healthcare, benefits, protections, and rights, are common experiences across many migrant worker populations internationally, collectively leading to higher risks of injury, illness, and death [107–109]. Therefore, adequate prevention strategies, improved healthcare access, and robust injury and death reporting, as well as inclusive methods to help surviving family members navigate bereavement entitlements, are of global relevance and should be explored further in future research.

## Data Availability

The data that support the findings of this study are available from the Office of the Chief Coroner of Ontario but restrictions apply to the availability of these data, which were used under a specific research agreement between the research team and the office, and so are not publicly available. Data are however available from the authors upon reasonable request and with permission of the Office of the Chief Coroner of Ontario.

## References

[1] Hayward SE, Deal A, Cheng C, Crawshaw A, Orcutt M, Vandrevala TF, Norredam M, Carballo M, Ciftci Y, Requena-Méndez A, Greenaway C (2021). Clinical outcomes and risk factors for COVID-19 among migrant populations in high-income countries: a systematic review. J Migr Health.

[2] Lusk JL, Chandra R (2021). Farmer and farm worker illnesses and deaths from COVID-19 and impacts on agricultural output. PLoS One.

[3] Van Barneveld K, Quinlan M, Kriesler P, Junor A, Baum F, Chowdhry A (2020). The COVID-19 pandemic: lessons on building more equal and sustainable societies. Labour (Rome).

[4] Fabreau GE, Holdbrook L, Peters CE, Ronksley PE, Attaran A, McBrien K (2022). Vaccines alone will not prevent COVID-19 outbreaks among migrant workers – The example of meat processing plants. Clin Microbiol Infect.

[5] Vosko LF, Spring C. COVID-19 outbreaks in Canada and the crisis of migrant farmworkers’ social reproduction: Transnational labour and the need for greater accountability among receiving states. J Int Migr Integr. 2021. Online ahead of print. 10.1007/s12134-021-00905-2 PMC8557264 34744516

[6] Caxaj CS, Cohen A, Colindres C, et al. Government COVID-19 guidelines gamble on the lives of migrant agricultural workers [Editorial]. Richmond Hill: Canadian Science Policy Centre, 2020 Apr. Available at https://sciencepolicy.ca/posts/government-covid-19-guidelines-gamble-on-the-lives-of-migrant-agricultural-workers/.

[7] Faraday F, Fudge J, Hanley J, McLaughlin J, Ramsaroop C, Tungohan E (2021). Migrant workers need priority access to the COVID-19 vaccine.

[8] Van Ingen T, Akingbola S, Brown KA, Daneman N, Buchan SA, Smith BT. Neighbourhood-level risk factors of COVID-19 incidence and mortality. MedRxiv. 2021 [Preprint]. https://www.medrxiv.org/content/10.1101/2021.01.27.21250618v1.full.pdf. 10.1371/journal.pone.0276507 PMC9584389 36264984

[9] Reitsma MB, Claypool AL, Vargo J, Shete PB, McCorvie R, Wheeler WH, Goldhaber-Fiebert JD. Racial/Ethnic Disparities In COVID-19 Exposure Risk, Testing, And Cases At The Subcounty Level In California: Study examines racial/ethnic disparities in COVID-19 risk, testing, and cases. Health Affairs. 2021;40(6):870–8. 10.1377/hlthaff.2021.00098 PMC8458028 33979192

[10] Reid A, Ronda-Perez E, Schenker MB (2021). Migrant workers, essential work, and COVID-19. Am J Ind Med.

[11] Grant T, Bailey I (2021). Five migrant farm workers have died since mid-March, four while in COVID-19 quarantine, advocacy group says.

[12] Migrant Worker Health Expert Working Group. “We need more than hollow words.” After third migrant agricultural worker death in Canada, experts denounce government inaction [Press Release]. Migrant Worker Health Project, 2020 June 23. Available at https://www.migrantworker.ca/we-need-more-than-hollow-words/.

[13] Global Compact for Migration. The global compact for safe, orderly and regular migration (a/RES/73/195). Morocco: United Nations Human Rights Office of the High Commissioner; 2018.

[14] World Health Organization (2018). Promoting the health of refugees and migrants: framework of priorities and guiding principles to promote the health of refugees and migrants.

[15] Moyce SC, Schenker M (2018). Migrant workers and their occupational health and safety. Annu Rev Public Health.

[16] Hansen E, Donohoe M (2003). Health issues of migrant and seasonal farmworkers. J Health Care Poor Underserved.

[17] Hu R, Shi L, Lee DC, Haile GP (2016). Access to and disparities in care among migrant and seasonal farm workers (MSFWs) at U.S. health centers. J Health Care Poor Underserved.

[18] Brian T (2021). Occupational fatalities among international Migrant workers: a global review of data sources.

[19] World Health Organization Health and Migration Programme (2022). Continuum of care for noncommunicable disease management during the migration cycle.

[20] Kilanowski JF (2017). Breadth of the socio-ecological model. J Agromedicine.

[21] Lee BC, Bendixsen C, Liebman AK, Gallagher SS (2017). Using the socio-ecological model to frame agricultural safety and health interventions. J Agromedicine.

[22] Kilanowski JF (2017). Breadth of the socio-ecological model. J Agromedicine.

[23] Centers for Disease Control and Prevention (2022). The socio-ecological model: a framework for prevention.

[24] Sallis JF, Owen N, Fisher EB, Glanz K, Rimer BK, Viswanath K (2008). Ecological models of health behavior. Health behavior and health education: theory, research, and practice.

[25] Employment and Social Development Canada (2022). Hire a temporary foreign agricultural worker.

[26] Employment and Social Development Canada (2022). Hire a temporary worker through the seasonal agricultural worker program: overview.

[27] Hennebry J (2012). Permanently temporary? agricultural migrant workers and their integration in Canada. Institute for research on public policy.

[28] McLaughlin J, Wells D, Mendiburo AD, Lyn A, Vasilevska B (2017). Temporary workers’, temporary fathers: transnational family impacts of Canada’s seasonal agricultural workers’ Program. Relat Ind.

[29] Statistics Canada (2022). Table 32-10-0221-01: countries of citizenship for temporary foreign workers in the agricultural sector.

[30] McLaughlin J, Hennebry J, Goldring L, Landolt P (2013). Pathways to precarity: structural vulnerabilities and lived consequences for migrant farmworkers in Canada. Producing and negotiating non-citizenship: precarious legal status in Canada.

[31] Basok T (2000). He came, he saw, he stayed: guest worker programmes and the issue of non-return. Int Migr.

[32] Walia H (2010). Transient servitude: Migrant labour in Canada and the apartheid of citizenship. Race Cl.

[33] Gesualdi-Fecteau D, Thibault A, Schivone N, Dufour C, Gouin S, Monjean N (2017). A story of debt and broken promises? The recruitment of Guatemalan migrant workers in Quebec. Que J Int Law.

[34] Gabriel C, Macdonald L (2018). After the international organization for migration: recruitment of Guatemalan temporary agricultural workers to Canada. J Ethn Migr Stud.

[35] Meyer C (2020). Migrant and undocumented workers plead for help during COVID-19. Canada’s National observer.

[36] Binford L (2009). From fields of power to fields of sweat: the dual process of constructing temporary migrant labour in Mexico and Canada. Third World Q.

[37] Caxaj CS, Cohen A, Marsden S. Supports for migrant farmworkers: Tensions in (In) access and (In) action. Int J Migr Health Soc Care. 2020;16(4):557–71.

[38] Caxaj CS, Cohen A, Colindres C. More of the same? Migrant agricultural workers' health, safety and legal rights in the COVID-19 context. Journal of Agricultural Food Systems and Community Development (In Press.)

[39] Otero G, Preibisch K (2015). Citizenship and precarious labour in Canadian agriculture.

[40] Juarez CE (2010). Sex, soccer and Saturday dance: free-time among the Mexican immigrants in Canada. Alteridades.

[41] McLaughlin J, Wells D, Mendiburo A, et al. ‘Temporary workers’, temporary fathers: Transnational family impacts of Canada’s seasonal agricultural worker program. Ind Relat. 2017;72(4):682–709.

[42] Colindres C, Cohen A, Caxaj CS. Migrant agricultural workers’ health, safety and access to protections: A descriptive survey identifying structural gaps and vulnerabilities in the interior of British Columbia, Canada. Int J of Environ Res Public Health. 2021;18(7):3696. 10.3390/ijerph18073696 PMC8037260 33916232

[43] Mayell S, McLaughlin J. Migrating to work at what cost? The cumulative health consequences of contemporary labour migration. In: Thomas F, ed. Handbook of migration and health. Cheltenham: Edward Elgar Publishing; 2016.

[44] Hennebry J, McLaughlin J, Preibisch K. Out of the loop:(In) access to health care for migrant workers in Canada. J Int Migr Integration. 2016;17(2):521–38.

[45] Mayell S. Up-rooted lives, deep-rooted memories: Stress and resilience among Jamaican agricultural workers in Southern Ontario. Hamilton: McMaster University MA thesis; 2016.

[46] Mayell S, McLaughlin J, Tew M. Migrant agricultural workers and Canada’s “not so universal” health care system: Lessons learned from an effort to improve access to health care in the province of Ontario. Presented at: The International Congress on Rural Health, Italy, 2015.

[47] McLaughlin J. Trouble in our fields: Health and human rights among Mexican and Caribbean migrant farm workers in Canada. Toronto: University of Toronto PhD thesis; 2009.

[48] McLaughlin J, Tew, M. Migrant farm worker health care: Unique strategies for a unique population. In: Arya N, Piggott T, eds. Under-served: Health determinants of Indigenous, inner-city, and migrant populations in Canada. Scholars’ Press, 2018. Anonymized for review.

[49] Perry JA (2018). Living at work and intra-worker sociality among migrant farm workers in Canada. J Int Migr Integr.

[50] Caxaj CS, Diaz L. Migrant workers’(non) belonging in rural British Columbia, Canada: storied experiences of marginal living. Int J Migr Health Soc Care. 2018;14(2):208–20.

[51] Caxaj CS, Cohen A. “I will not leave my body here”: Migrant farmworkers’ health and safety amidst a climate of coercion. Int J Environ Res Public Health. 2019;16(15):2643. 10.3390/ijerph16152643 PMC6695666 31344912

[52] Basok T, Belanger D, Rivas E (2014). Reproducing deportability: Migrant agricultural workers in South-Western Ontario. J Ethn Migr Stud.

[53] Casey R, Tucker E, Vosko LF (2019). Enforcing employment standards for temporary migrant agricultural workers in Ontario, Canada: exposing underexplored layers of vulnerability. Int J Comp Labour Law Ind Relat.

[54] Cohen A, Caxaj CS. Bodies and borders: migrant women farmworkers and the struggle for sexual and reproductive justice in British Columbia, Canada. Alternate Routes. J Crit Soc Res. 2018;29.

[55] Caxaj CS, Cohen A. Relentless Border Walls: Challenges of Providing Services and Supports to Migrant Agricultural Workers in British Columbia. Canadian Ethnic Stud. 2021;53(2):41–67.

[56] McLaughlin J, Tew M, Huesca E. Compounded vulnerabilities and creative strategies: Occupational health of temporary foreign agricultural workers. In: Premji S, ed. Sick and tired: Health and safety inequalities. Halifax: Fernwood; 2018.

[57] Cole DC, McLaughlin J, Hennebry J, et al. Precarious patients: A mixed methods study of health professionals’ perspectives on providing care to Mexican and Jamaican migrants in Canada’s Seasonal Agricultural Worker Program. Rural and Remote Health. 2019;19(4):5313. 10.22605/RRH5313 31785605

[58] Mema S, Frosst G, Hanson K, Yates C, Anderson A, Jacobsen J (2021). Foodborne and animal contact disease outbreaks: COVID-19 outbreak among temporary foreign workers in British Columbia, march to may 2020. Can Commun Dis Rep.

[59] Migrant Worker Health Expert Working Group. Recommendations for overcoming health challenges faced by migrant agricultural workers during the COVID-19-virus pandemic. Migrant Worker Health Project, 2020.

[60] Basok T, George G (2020). “We are part of this place, but I do not think I belong.” Temporariness, social inclusion and belonging among migrant farmworkers in Southwestern Ontario. Int Migr.

[61] Hennebry J, Caxaj CS, McLaughlin J, et al. Coronavirus: Canada stigmatizes, jeopardizes essential migrant workers [Editorial]. The Conversation; 2020. Available at https://theconversation.com/coronavirus-canada-stigmatizes-jeopardizes-essential-migrant-workers-138879.

[62] Preibisch K (2004). Migrant agricultural workers and processes of social inclusion in rural Canada: Encuentros and desencuentros. Can J Lat Am Caribb Stud.

[63] Orkin AM, Lay M, McLaughlin J, Schwandt M, Cole D (2014). Medical repatriation of migrant farm workers in Ontario: a descriptive analysis. CMAJ Open.

[64] Rodgers A (2018). Envisioning justice for migrant workers: a legal needs assessment.

[65] Weiler AM, Otero G, Wittman H (2016). Rock stars and bad apples: moral economies of alternative food networks and precarious farm work regimes. Antipode.

[66] Vosko LF. Blacklisting as a modality of deportability: Mexico's response to circular migrant agricultural workers' pursuit of collective bargaining rights in British Columbia, Canada. J Ethnic Migr Stud. 2016;42(8):1371–87.

[67] Auditor General of Canada (2021). Report 13: health and safety of agritcultural temporary foreign workers in Canada during the COVID-19 pandemic.

[68] Marsden S, Tucker E, Vosko LF. Flawed by design? A case study of federal enforcement of migrant workers' labour rights in Canada. Canadian Labour Employ Law J. 2021;23(1):71–102.

[69] McLaughlin J, Hennebry J, Haines T. Paper versus practice: occupational health and safety protections and realities for temporary foreign agricultural workers in Ontario. Perspectives interdisciplinaires sur le travail et la santé. 2014;16(2).

[70] Haley E, Caxaj S, George G, Hennebry J, Martell E, McLaughlin J. Migrant farmworkers face heightened vulnerabilities during COVID-19. J Agric Food Sys Community Dev. 2020;9(3):35–9.

[71] Tucker EM, Marsden S, Vosko LF. Federal enforcement of migrant workers’ labour rights in Canada: A research report [Pre-print]. Toronto: Osgoode Digital Commons, Articles & Book Chapters 2795; 2020.

[72] Vosko LF. Legal but deportable: Institutionalized deportability and the limits of collective bargaining among participants in Canada’s seasonal agricultural workers program. ILR Rev. 2018;71(4):882–907.

[73] Vosko LF. Disrupting deportability: Transnational workers organize. Ithaca: Cornell University Press; 2019. p. 192.

[74] Sargeant M, Tucker E (2019). Layers of vulnerability in occupational safety and health for migrant workers: case studies from Canada and the UK. Policy Prac Health Saf.

[75] Caxaj CS, Cohen A, Buffam B, et al. Borders and boundaries in the lives of migrant agricultural workers: Towards a more equitable health services approach. Witness: Can J Crit Nursing Discourse. 2020;2(2):92–103.

[76] Magalhaes L, Carrasco C, Gastaldo D (2010). Undocumented migrants in Canada: a scope literature review on health, access to services, and working conditions. J Immigr Minor Health.

[77] Hanley J, Oxman-Martinez J, Lacroix M, Gal S (2006). The “deserving” undocumented?: Government and community response to human trafficking as a labour phenomenon. Labour Cap Soc.

[78] Salami B, Hervieux E, Dorow S, Okeke-Ihejirika P (2019). Intensified exploitation and mental stress as impacts of changes to the temporary foreign worker program in Alberta, Canada. Glob Soc Welf.

[79] Zwaigenbaum J, Salami B, Tulli M (2021). The ongoing exploitation of temporary foreign workers.

[80] Neergaard MA, Olesen F, Andersen RS, Sondergaard J (2009). Qualitative description - the poor cousin of health research?. BMC Med Res Methodol.

[81] Sullivan-Bolyai S, Bova C, Harper D (2005). Developing and refining interventions in persons with health disparities: the use of qualitative description. Nurs Outlook.

[82] Morawska L, Milton DK. It is time to address airborne transmission of coronavirus disease 2019 (COVID-19). Clin Infect Dis. 2020;71(9):2311–3. 10.1093/cid/ciaa939 PMC7454469 32628269

[83] Rabaan AA, Al-Ahmed SH, Al-Malkey M, Al-Malkey M, Alsubki R, Ezzikpouri S, Al-Hababi FH (2021). Airborne transmission of SARS-CoV-2 is the dominant route of transmission: droplets and aerosols. Infez Med.

[84] Hennebry J. The road taken: temporary labour migration in Canada’s immigration system. In: Samy Y, Duncan H, editors. International affairs and Canadian migration policy. Cham: Palgrave Macmillan; 2020.

[85] Basok T, Bélanger D, Rivas E (2014). Reproducing deportability: Migrant agricultural workers in South-Western Ontario. J Ethn Migr Stud.

[86] Vosko LF. Disrupting Deportability. In: Disrupting deportability. London: Cornell University Press; 2019.

[87] Wickramasekara, P. Circular migration: A triple win or a dead end. Global Union Research Network Discussion Paper. Geneva: International Labour Office; 2011. Retrieved July 5 2022 from https://citeseerx.ist.psu.edu/viewdoc/download?doi=10.1.1.978.2066&rep=rep1&type=pdf.

[88] Hanley J, Ives N, Lenet J, Hordyk SR, Walsh CA, Soltane SB (2019). Migrant women’s health and housing insecurity: an intersectional analysis. Int J Migr Health Soc Care.

[89] Horgan M, Liinamaa S (2017). The social quarantining of migrant labour: everyday effects of temporary foreign worker regulation in Canada. J Ethn Migr Stud.

[90] Preibisch K, Hennebry J (2011). Temporary migration, chronic effects: the health of international migrant workers in Canada. CMAJ.

[91] Farha L. Special Rapporteur on the right to adequate housing. Guidelines for the implementation of the right to adequate housing. Geneva: United Nations Human rights Office of the High Comissioner; 2019. Retrieved July 5 from https://documents-dds-ny.un.org/doc/UNDOC/GEN/G19/353/90/PDF/G1935390.pdf?OpenElement.

[92] Robillard C, McLaughlin J, Cole DC, Vasilevska B, Gendron R (2018). “Caught in the same webs” – service providers’ insights on gender-based and structural violence among female temporary foreign workers in Canada. J Int Migr Integr.

[93] Hennebry J, Hari KC. Quarantined! Xenophobia and migrant workers during the COVID-19 pandemic. Geneva: International Organization for Migration (IOM); 2020. Retrieved July 5 2022 from https://publications.iom.int/system/files/pdf/quarantined.pdf.

[94] Hjalmarson E. Sentenced for the season: Jamaican migrant farmworkers on Okanagan orchards. Race Class. 2021;18:03063968211054856.

[95] Johnstone MJ, Kanitsaki O (2010). The neglect of racism as an ethical issue in health care. J Immigr Minor Health.

[96] Salihu HM, Wilson RE, King LM, Marty PJ, Whiteman VE (2015). Socio-ecological model as a framework for overcoming barriers and challenges in randomized control trials in minority and underserved communities. Int J MCH AIDS.

[97] Daley E, Alio A, Anstey EH, Chandler R, Dyer K, Helmy H (2011). Examining barriers to cervical cancer screening and treatment in Florida through a socio-ecological lens. J Community Health.

[98] Basok T, Belanger D (2016). Migration management, disciplinary power, and performances of subjectivity: agricultural migrant workers’ in Ontario. Can J Sociol.

[99] Basok T, Bélanger D, Rivas E (2014). Reproducing deportability: migrant agricultural workers in South-Western Ontario. J Ethn Migr Stud.

[100] McLaughlin J, Hennebry J. Pathways to precarity: structural vulnerabilities and lived consequences for migrant farmworkers in Canada. In Goldring, L., P. Landolt, editors. Producing Negotiating Non-Citizenship: Precarious legal status in Canada. Toronto: University of Toronto; 2013. p. 175–94.

[101] Alahmad B, AlMekhled D, Odeh A, Albloushi D, Gasana J (2021). Disparities in excess deaths from the COVID-19 pandemic among migrant workers in Kuwait. BMC Public Health.

[102] Paremoer L, Nandi S, Serag H, Baum F (2021). Covid-19 pandemic and the social determinants of health. BMJ.

[103] Hannon M (2022). Tenant rights for employer-provided farmworker housing. Seattle J Soc Justice.

[104] Lee JG, LePrevost CE, Harwell EL, Bloss JE, Cofie LE, Wiggins MF, Firnhaber GC (2020). Coronavirus pandemic highlights critical gaps in rural internet access for migrant and seasonal farmworkers: a call for partnership with medical libraries. J Med Libr Assoc.

[105] Li J, Liu Z (2018). Housing stress and mental health of migrant populations in urban China. Cities.

[106] Perocco F. The coronavirus crisis and the consequences of COVID-19 Pan-Syndemic on racial health inequalities and on migrants. In Della Puppa, F. D. & Guiliana, S. editors Stuck and Exploited. Refugees and asylum seekers in Italy between exclusion, discrimination and struggles. Venice, Italy. Ca' Foscari: 2021:239.

[107] Alahmad B, AlMekhled D, Odeh A, Albloushi D, Gasana J (2021). Disparities in excess deaths from the COVID-19 pandemic among migrant workers in Kuwait. BMC Public Health.

[108] McLaughlin. Migrating to Work at what Cost?: The Cumulative Health Consequences of Contemporary Labour Migration. Handbook of Migration and Health, Felicity Thomas (ed.), Cheltenham, UK: Edward ElgarPublishing; 2016. p. 230–52.

[109] Yea S (2022). The produced injured: locating workplace accidents amongst precarious migrant workmen in Singapore. Soc Sci Med.

